# Clustering of chronic hepatitis B screening intentions in social networks of Moroccan immigrants in the Netherlands

**DOI:** 10.1186/s12889-020-8438-x

**Published:** 2020-03-17

**Authors:** Nora Hamdiui, Vincent Buskens, Jim E. van Steenbergen, Mirjam E. E. Kretzschmar, Luis E. C. Rocha, Anna E. Thorson, Aura Timen, Albert Wong, Maria van den Muijsenbergh, Mart L. Stein

**Affiliations:** 1 National Coordination Centre for Communicable Disease Control, Centre for Infectious Disease Control, National Institute for Public Health and the Environment, RIVM/LCI, Postbus 1 (Postbak 13), 3720 BA Bilthoven, The Netherlands; 2 Julius Center for Health Sciences and Primary Care, University Medical Center Utrecht, Utrecht University, Utrecht, The Netherlands; 3 Department of Sociology/ICS, Utrecht University, Utrecht, The Netherlands; 4 Centre for Infectious Diseases, Leiden University Medical Centre, Leiden, The Netherlands; 5 Centre for Infectious Disease Control, National Institute for Public Health and the Environment, Bilthoven, The Netherlands; 6 Department of Economics & Department of Physics and Astronomy, Ghent University, Ghent, Belgium; 7 Department of Public Health Sciences, Karolinska Institutet, Stockholm, Sweden; 8 Athena Institute for Research on Innovation and Communication in Health and Life Sciences, VU University Amsterdam, Amsterdam, The Netherlands; 9 Department of Statistics, Informatics and Modeling, National Institute for Public Health and the Environment, Bilthoven, The Netherlands; 10 Pharos: Dutch Centre of Expertise on Health Disparities, Program Prevention and Care, Utrecht, The Netherlands; 11 Radboud University Medical Center, Radboud Institute for Health Sciences , Department of Primary and Community Care, Nijmegen, The Netherlands

**Keywords:** Social networks, Hepatitis B, Screening, Intention, Moroccan immigrants, Netherlands, Respondent-driven sampling

## Abstract

**Background:**

Early detection, identification, and treatment of chronic hepatitis B through screening is vital for those at increased risk, e.g. born in hepatitis B endemic countries. In the Netherlands, Moroccan immigrants show low participation rates in health-related screening programmes. Since social networks influence health behaviour, we investigated whether similar screening intentions for chronic hepatitis B cluster within social networks of Moroccan immigrants.

**Methods:**

We used respondent-driven sampling (RDS) where each participant (“recruiter”) was asked to complete a questionnaire and to recruit three Moroccans (“recruitees”) from their social network. Logistic regression analyses were used to analyse whether the recruiters’ intention to request a screening test was similar to the intention of their recruitees.

**Results:**

We sampled 354 recruiter-recruitee pairs: for 154 pairs both participants had a positive screening intention, for 68 pairs both had a negative screening intention, and the remaining 132 pairs had a discordant intention to request a screening test. A tie between a recruiter and recruitee was associated with having the same screening intention, after correction for sociodemographic variables (OR 1.70 [1.15–2.51]).

**Conclusions:**

The findings of our pilot study show clustering of screening intention among individuals in the same network. This provides opportunities for social network interventions to encourage participation in hepatitis B screening initiatives.

## Background

Chronic hepatitis B (HBV) is a major global health problem. If untreated, it may put people at an increased risk for chronic sequelae including liver cirrhosis and fibrosis, leading to premature death. HBV prevalence is the highest in the World Health Organization (WHO) regions Western Pacific and Africa. Here, 6.2 and 6.1% of the population is chronically infected, respectively [1].

Although the Netherlands is a low-endemic country for chronic HBV (prevalence: 0.1%) [2], several risk groups have a significantly higher prevalence of HBV carriage, the largest of which being immigrants from intermediate or high endemic countries [3, 4]. Of these immigrants, an estimated 5.4% is chronically infected [2, 5]. While a free of charge vaccination programme targeting behavioural high-risk groups has been introduced in 2002 [6], universal HBV vaccination has only been introduced in the Netherlands in 2011, with four vaccine doses given at the ages of 2, 3, 4, and 11 months [7]. Considering the predominance of mother-to-child HBV transmission among immigrants born in endemic countries, many immigrants arriving in the Netherlands could already be infected and for them vaccination has negligible benefit. Therefore, screening for the hepatitis B surface antigen (HBsAg) as sign of chronic infection is the only option. In November 2016, the Dutch Health Council recommended HBsAg-screening for first-generation immigrants originating from intermediate (2–7%) or high (≥ 8%) HBV endemic countries [8]. This screening for HBsAg aims to detect unnoticed asymptomatic chronically infected individuals for either immediate treatment or monitoring, and to prevent further transmission [9]. Although this screening is recommended for immigrants originating from countries with a HBV endemicity of 2% or higher, it proved to be cost-effective for those originating from countries with a HBV prevalence of 0.41% or higher [10]. Based on three small regional Dutch studies, the prevalence of chronic HBV among Moroccans, who form the second largest immigrant group in the Netherlands, is low (0.54% [95% CI 0.01–1.07]) but within the range targeted for screening [11]. We chose to target Moroccan immigrants in our pilot study, because of the proven cost-effectiveness and having our existing infrastructure [12, 13]. Following the guidelines of Statistics Netherlands, we also define first-generation Moroccan immigrants as individuals born in Morocco and having at least one parent born in Morocco, and second-generation Moroccan immigrants as individuals born in the Netherlands and having at least one parent born in Morocco [14].

The Council recommended two strategies to screen these first-generation immigrants: [1] individual case finding by general practitioners (GP), and [2] local screening programmes. Both strategies start with an HBV test (costing EUR 25 (2019)). Since the Dutch health insurance is organised with a compulsory annual amount (“front-end deductible”) of EUR 385 (2019) which you have to pay for health services before your health insurance begins to pay, the HBV test is not refundable for those for whom this threshold has not been reached yet with other health care costs [12]. However, Moroccan immigrants show lower participation rates in health-related screening programmes compared to indigenous and other immigrant populations [15–18]. Previous qualitative and quantitative research showed that the main reasons for this nonparticipation in HBV screening initiatives were shame and stigma, fatalism (i.e. an attitude of resignation in the face of some future event or events which are thought to be inevitable), and the perceived burden of participating in such a screening [12, 13].

However, not only characteristics of a single individual are important, as research showed that health behaviour is also influenced by the individuals’ social contacts [19–21]. For example, an American study found an increase in breast cancer screening participation among women whose sisters were screened and in colorectal screening participation if spouses were screened [22]. Higher levels of the intention to screen for cardiovascular disease were observed among Mexican-Americans when participants had at least one older-generation peer who encouraged screening [23]. Encouragement by family and/or friends and the perception that screening was normative were also found to be predictive for having a mammogram among American women [24]. Furthermore, studies from the United States suggest that obesity, smoking, and happiness spread in social networks through social influence [19, 20, 25]. For Moroccan immigrants specifically, scientific evidence points at the crucial role of an individuals’ social network for coping with perceived ethnic discrimination, the use of psychosocial services, and pregnancy-related health behaviour [26–28].

However, the majority of studies investigated the role of social contacts in preventive health behaviour using egocentric data, i.e., responses of participants who were sampled independently of one another. In most cases with egocentric approaches, researchers cannot contact participant’s peers directly and must rely on what participants report about their social connections’ characteristics [29]. There is potentially relevant information that participants simply do not know about their social connections, such as intentions to participate in screening. By contrast, few studies collected saturated network data, which includes information on all nodes and connections within a specific population. Saturated approaches can be costly, time consuming, and thus limited to very small populations [29].

In this pilot study, we used respondent-driven sampling (RDS) to sample social contacts. RDS is a variant of chain-referral sampling, which was originally developed to study hard-to-reach populations and to calculate unbiased population estimates [30]. We used RDS to reach immigrants and for social network analysis where a tie between two individuals is the unit of analysis, instead of the individual itself [29, 30]. This enabled us to address our hypothesis, namely that similar HBV screening intentions among Moroccan immigrants living in the Netherlands are clustered within their close social networks (i.e. family, friends, and workmates sharing the same positive or negative screening intention) due to a strong sense of community and trust within the group. The collection and analyses of empirical network data are important first steps to help future studies in selecting appropriate network interventions to encourage participation in HBV screening initiatives [31].

## Methods

### Study population

In 2018, there were 396,539 Moroccans in the Netherlands of which 169,018 first and 227,521 second generation (as defined by Statistics Netherlands) [32]. The children of this second generation are defined as non-immigrant and are, thus, not registered as third-generation immigrant. Of all first-generation Moroccan immigrants in the Netherlands, 21% live in Amsterdam, 12% in Rotterdam, 8% in Utrecht, and 8% in the Hague. Some medium-sized municipalities, including Gouda, Almere, Leiden, Haarlem, Eindhoven, and Tilburg, are also cities where relatively large numbers of Moroccans of the first generation live [33].

### Study design

From November 2016 to February 2017, first-generation Moroccan immigrants and their children and grandchildren were recruited throughout the Netherlands using offline and online respondent-driven sampling (RDS) to identify determinants of one’s intention to participate in screening for chronic HBV, of which methods and results were described earlier [30]. Although children of second-generation immigrants are non-immigrants, we included both children and grandchildren of first-generation Moroccan immigrants. In immigrant families, both children and grandchildren play an important social role for - and have a close relationship with - their parents and grandparents. They act as instructors, models, and interpreters, and provide financial, social, and/or emotional support to their parents and grandparents. Therefore, both children and grandchildren are an important group to consider when studying health behaviour of first-generation immigrants [34, 35], since they frequently act as brokers for their parents or grandparents in contact with the Dutch healthcare [36].

RDS starts with a convenience sample of the target population (so-called “seeds”). We recruited seeds offline, at community venues (such as community centres, day care centres, and mosques) and by approaching interest groups and civil support foundations in the aforementioned regions. Here, small groups of only men or only women regularly came together for cooking workshops, Dutch language courses, and Quran readings. Online, seeds were recruited through advertisements on Moroccan-Dutch forums, Facebook, Instagram, the website of the Dutch National Institute for Public Health and the Environment (RIVM), and a Moroccan-Dutch website [37]. Both offline and online questionnaires were filled in by the participants themselves with an option of translation by - or getting assistance - of a Berber-speaking researcher. Seeds (“recruiters” representing wave zero) were asked to complete a questionnaire and invite at least three Moroccans from their social network (“recruitees”) to complete the same questionnaire. Initial recruitees (representing wave one) were also asked to recruit others. Therefore, they became “recruiters” too, which led to wave two, and so on. Each recruiter, who recruited at least three recruitees, received one gift coupon. The values of these coupons were EUR 5, when our study started, and were raised to EUR 10, and EUR 25, in later stages of the study in order to increase recruitment. The value was increased to stimulate peer-recruitment among all new participants. Invitations containing unique and anonymous codes (more details can be found in a previous publication [12]) enabled us to follow who invited whom and to visualise and analyse their social networks. Eligibility criteria included: 1) age ≥ 16 years; 2) born in Morocco and having at least one parent born in Morocco, *or* born in the Netherlands and having at least one (grand-) parent born in Morocco; and 3) residing in the Netherlands.

The link between each recruiter with his/her recruitee was defined as a “*tie”*. A “tie” is further distinguished into “***RDS ties***” and “***venue ties***”:

- Since an invitation must be physically transferred from the recruiter to the recruitee following RDS, these links are further referred to as “***RDS ties***”.
- Since the majority of offline-recruited participants entailed small groups that regularly came together for a variety of activities, we assumed that all participants recruited at one community venue knew each other and thus were connected, which resulted in additional ties per community venue (further referred to as “***venue ties***”).

“RDS ties” and “venue ties” were both defined as “*having a tie*”. We tested whether the assumption that these two types of ties have similar effects is reasonable.

### Questionnaire

We developed and used a questionnaire in Dutch, in which questions were based on a compilation of the Health Belief Model (HBM), the Theory of Planned Behaviour (TPB), and Betancourt’s Model of Culture and Behaviour [38] following earlier studies that investigated HBV screening intention among the Turkish-Dutch population [39]. The HBM assumes that a subject is more likely to take a “health action” whenever s/he perceives 1) the disease as serious, 2) herself or himself susceptible to the disease, 3) benefits of the health action, 4) limited barriers to take the health action, 5) self-efficacy in relation to the health action, and whenever 6) s/he receives a cue to take the health action. According to the TPB, intention reflects a person’s readiness to perform a certain health behaviour or action, explained by attitude, subjective norm, and perceived behavioural control. Betancourt’s Model of Culture and Behaviour includes culture to explain its effect on health behaviours. The questionnaire focused on the predominance of mother-to-child HBV transmission and not on other possible transmission routes, such as sexual contact. This was to avoid feelings of shame and stigma, which were found to exist in previous studies [12, 13]. To classify identity, a question regarding mother tongue was included: Dutch, Moroccan-Arabic, Berber, Modern Standard Arabic, and/or other. Those who reported to be speakers of Berber were defined as having a Berber identity, whereas those who reported to be speakers of Moroccan-Arabic and/or Modern Standard Arabic and not Berber were defined as having a Moroccan-Arabic identity. This grouping was done since these languages represent (to some extent) two social subgroups within the Moroccan community. In the questionnaire, our outcome variables (i.e. intention to request a test and intention to participate) were measured using the questions: “Imagine, you go the GP tomorrow. Would you request a HBV test?” and “Imagine, your GP advises you to have yourself tested for HBV. Would you participate in HBV screening if you would have to pay EUR 70 for this test?”. We will further refer to these outcome measures as ‘Intention request’ and ‘Intention 70’. All variables measured by the questionnaire are depicted in Table S1.

### Statistical analysis

To study the distribution of similar intentions within the sampled social networks, we chose to analyse dyads (i.e. pairs of individuals), which are the smallest type of social structure in which an individual can be embedded. To obtain the dyad as the level of analysis, we first defined the set of dyads as the set of all possible pairs of participants in the sample, i.e. we constructed a set of n(n-1)/2 dyads. Then, we constructed the variable “tie”. This variable is coded one for pairs of participants who have either a RDS or a venue tie. The variable is coded zero for all other pairs of participants in the dataset. For each pair, we checked whether they had the same intention for requesting a HBV test on own initiative (outcome one, ‘Intention request’) and the same intention to participate in HBV screening for non-refundable costs of EUR 70 (outcome two, ‘Intention 70’). Logistic regression was used to analyse whether tie equal to one increases the likelihood that in a pair of individuals both have the same intention.

#### Non-hierarchical structure

**Fig. 1:**
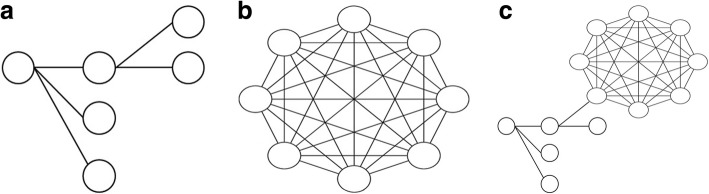
(**a**). Recruitment tree with only RDS ties, (**b**). Recruitment tree with only venue ties, (**c**). Recruitment tree with both RDS ties and venue ties

A logistic regression model assumes that observations are independent of each other, which is not the case in our sample, since participants were involved in multiple pairs (i.e. multiple times as recruiter, or as both recruiter and recruitee) and participants were also directly or indirectly linked in recruitment trees (see Fig. 1 a-c). If only RDS ties were present, a recruitment tree consisted of the seed (wave zero) and all consecutive waves with participants who all share this seed (see Fig. 1 a). If only venue ties were present, a recruitment tree consisted of all participants recruited at one community venue being connected to each other (see Fig. 1 b). If both types of ties were present, a recruitment tree consisted of the seed and its consecutive waves, with participants connected to each other (as a group) representing one community venue (see Fig. 1 c). These recruitment trees are further referred to as “clusters”.

**Fig. 2:**
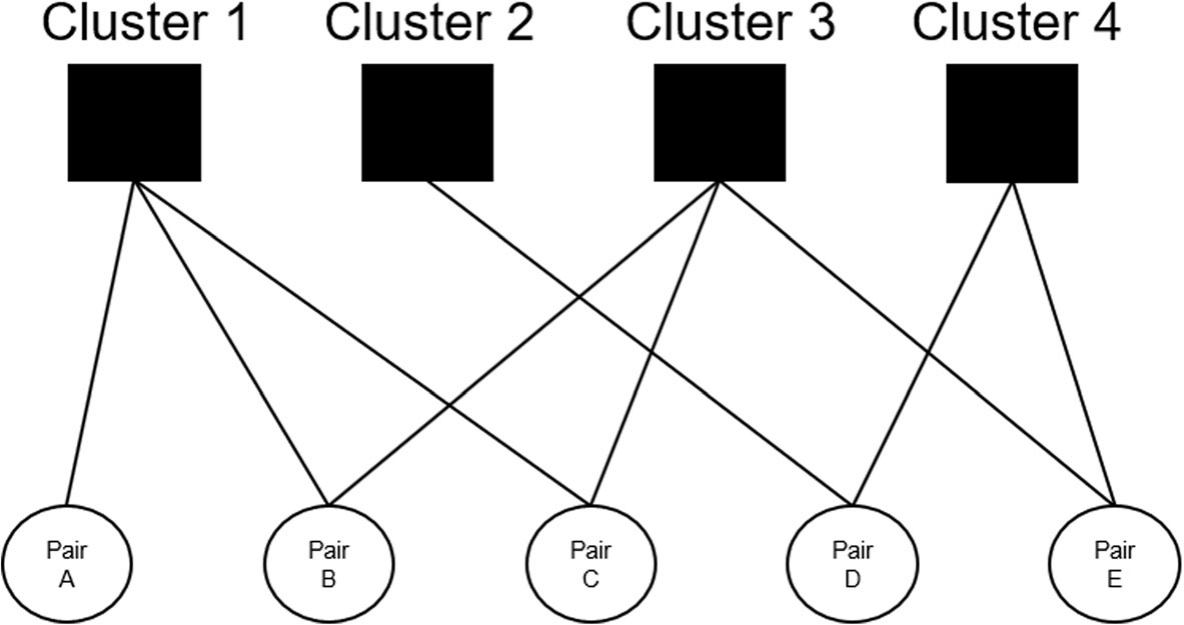
Data structure of clusters and pairs. By using robust standard errors adapted for multi-way clustering, pairs with at least one participant in the same cluster (see pair A and pair B for example) are considered dependent observations. Pairs without participants in the same clusters (see pair A and pair E for example) are considered independent observations

This creates a non-hierarchical (i.e. multi-way) nesting structure of observations of pairs each nested in one or two clusters (see Fig. 2). We controlled for this non-hierarchical clustering by using robust standard errors adapted for multi-way clustering as suggested by Cameron et al. [40]. With this method, pairs nested in overlapping clusters are considered dependent observations, whereas pairs nested in different clusters are considered independent observations. Thus, pairs are considered dependent observations if at least one node of each pair is present within the same cluster.

#### Logistic regression models

We constructed four models for each of the two outcomes. First, the variable “tie” was included as the single independent variable (Model I). Thereafter, we included the second independent binary variable “type of tie”, with the categories: close family relationship yes/no (i.e. a family member or partner living in the same household) (Model II). Doing so, we investigated whether the association between tie and having the same screening intention differed for the type of tie studied. Subsequently, in Model III, we added several sociodemographic variables, namely: having the same gender (with the following categories: woman-woman versus man-man and man-woman versus man-man), mean age, age difference, same country of birth (with the following categories: Netherlands-Netherlands versus Morocco-Morocco and Netherlands-Morocco versus Morocco-Morocco), and the same educational level. The mean value of the pairs’ educational level was added by taking the mean value of the coding values of “educational level”. With a mean value of three for example, one participant may have an educational level coded as two and the other one as four. The participant’s attitude towards fatalism in the context of screening was also included, since it incorporates, to some extent, one’s individual religious interpretation and its influence on screening intention, which we believe is an important individual characteristic to include in the model [41]. Prior to this study, determinants of individual screening intention were studied qualitatively [13] and quantitatively [12]. The five most important determinants of individual screening intention (wanting clarity, fatalism, not having symptoms, self-efficacy, and risk perception) (found in this previous study [12]) were added in the final logistic regression model (Model IV) to assess whether the underlying determinants of individual screening intention are (also) concurrent between participants with a tie.

In Models III and IV, we also constructed variables at the dyad level for each sociodemographic variable and each determinant of individual screening intention. Two covariates were included for each determinant. As an example, for the sociodemographic variable “educational level” we included “having the same educational level” (1 = yes / 0 = no) and the mean educational level. This enabled us to distinguish whether a pair has the same screening intention 1) because of having the same educational level *or* 2) because of the educational level itself. In other words, it may be possible that having the *same* educational level is not associated with having the same screening intention, but that a *high* education level is associated with having the same *positive* screening intention. For age, we included the difference in age between pairs to incorporate its influence on having the same screening intention.

To determine associations of intention for each combination of pairs specifically (discordant, both positive, or both negative), multinomial logistic regression analyses would be needed. For the interpretability of our results, however, we chose to repeat Models I to IV, but then with the dependent variable regrouped into “having the same *positive* intention” (1 = yes / 0 = no) and “having the same *negative* intention” (1 = yes / 0 = no).

All analyses were conducted two-tailed, significance tests with α = 0.05, using R version 3.4.0 and STATA version 14.2.

## Results

### Study participants and pairs with a tie

The study population was composed of 379 Moroccan immigrants, of which 156 (41.2%) were recruited offline and 223 (58.8%) online (see Table 1). Of these participants, 59.5% were seeds and 40.5% were recruited by their social contacts (recruitees). By using online RDS, we mainly reached younger second-generation immigrants and their children, with a higher educational level, compared to those recruited offline. Those recruited offline had more willingness to participate in screening for non-refundable costs of EUR 70 compared to online-recruited participants. Of the total study population, 269 (71%) would test themselves if a friend recommends having a HBV test.

**Table 1:** Sample characteristics stratified for offline/online recruitment, *n* = 379

Characteristic	Offline-recruited participants *(n = 156, 41.2%)*	Online-recruited participants *(n = 223, 58.8%)*	Total *(n = 379)*
Will test myself if friend recommends			
Yes	106 (67.9)	163 (73.1)	269 (71.0)
No	19 (12.2)	16 (7.2)	35 (9.2)
I do not know	29 (18.6)	24 (10.8)	53 (14.0)
*Missing value*	2 (1.3)	20 (9.0)	22 (5.8)
Intention request			
Yes/probably yes	78 (50.0)	109 (48.9)	187 (49.3)
No/probably not	74 (47.4)	94 (42.2)	168 (44.3)
*Missing value*	4 (2.6)	20 (9.0)	24 (6.3)
Intention 70			
Yes/probably yes	83 (53.2)	84 (37.7)	167 (44.1)
No/probably not	59 (37.8)	117 (52.5)	176 (46.4)
*Missing value*	14 (9.0)	22 (9.9)	36 (9.5)
Country of birth			
Morocco	110 (70.5)	83 (37.2)	193 (50.9)
The Netherlands	46 (29.5)	140 (62.8)	186 (49.1)
*Missing value*	0 (0)	0 (0)	0 (0)
Moroccan-Arabic or Berber identity			
Arabic	61 (39.1)	90 (40.4)	151 (39.8)
Berber	94 (60.3)	133 (59.6)	227 (59.9)
*Missing value*	1 (0.6)	0 (0)	1 (0.3)
Gender			
Man	52 (33.3)	71 (31.8)	123 (32.5)
Woman	104 (66.7)	152 (68.2)	256 (67.5)
*Missing value*	0 (0)	0 (0)	0 (0)
Age group			
16–25 years	22 (14.1)	64 (28.7)	86 (22.7)
26–35 years	16 (10.3)	61 (27.4)	77 (20.3)
36–45 years	34 (21.8)	60 (26.9)	94 (24.8)
46–55 years	35 (22.4)	29 (13.0)	64 (16.9)
56–65 years	25 (16.0)	6 (2.7)	31 (8.2)
66 years and older	12 (7.7)	2 (0.9)	14 (3.7)
*Missing value*	12 (7.7)	1 (0.4)	13 (3.4)
Educational level			
No official education or primary school	51 (32.7)	15 (6.7)	66 (17.4)
Secondary school	34 (21.8)	41 (18.4)	75 (19.8)
Vocational education	36 (23.1)	61 (27.4)	97 (25.6)
Higher education	32 (20.5)	103 (46.2)	135 (35.6)
*Missing value*	3 (1.9)	3 (1.3)	6 (1.6)
Speaking Dutch (SR)			
Yes	145 (92.9)	221 (99.1)	366 (96.6)
No	10 (6.4)	2 (0.9)	12 (3.2)
*Missing value*	1 (0.6)	0 (0)	1 (0.3)
Knowledge on HBV			
No	70 (44.9)	82 (36.8)	152 (40.1)
Limited	57 (36.5)	105 (47.1)	162 (42.7)
Sufficient	29 (18.6)	36 (16.1)	65 (17.2)
*Missing value*	0 (0)	0 (0)	0 (0)
HBV in family or friends			
Yes	43 (27.6)	34 (15.2)	77 (20.3)
No	98 (62.8)	156 (70.0)	254 (67.0)
I do not know	15 (9.6)	33 (14.8)	48 (12.7)
*Missing value*	0 (0)	0 (0)	0 (0)
Tested for HBV (SR)			
Yes	30 (19.2)	49 (22.0)	79 (20.8)
No	110 (70.5)	149 (66.8)	259 (68.3)
I do not know	15 (9.6)	25 (11.2)	40 (10.6)
*Missing value*	1 (0.6)	0 (0)	1 (0.3)
Vaccinated against HBV (SR)			
Yes	42 (26.9)	73 (32.7)	115 (30.3)
No	61 (39.1)	47 (21.1)	108 (28.5)
I do not know	53 (34.0)	103 (46.2)	156 (41.2)
*Missing value*	0 (0)	0 (0)	0 (0)
Who invited you for the questionnaire?			
Family or partner	46 (29.5)	74 (33.2)	120 (31.7)
Friend, acquaintance, or workmate	40 (25.6)	60 (26.9)	100 (26.4)
Researcher of the RIVM	32 (20.5)	19 (8.5)	47 (12.4)
Via a message on a website	0 (0)	58 (26.0)	58 (15.3)
Someone else	37 (23.7)	10 (4.5)	51 (13.5)
*Missing value*	1 (0.6)	2 (0.9)	3 (0.8)

Data are reported as number of participants (%)

*SR* Self-reported

**Fig. 3:**
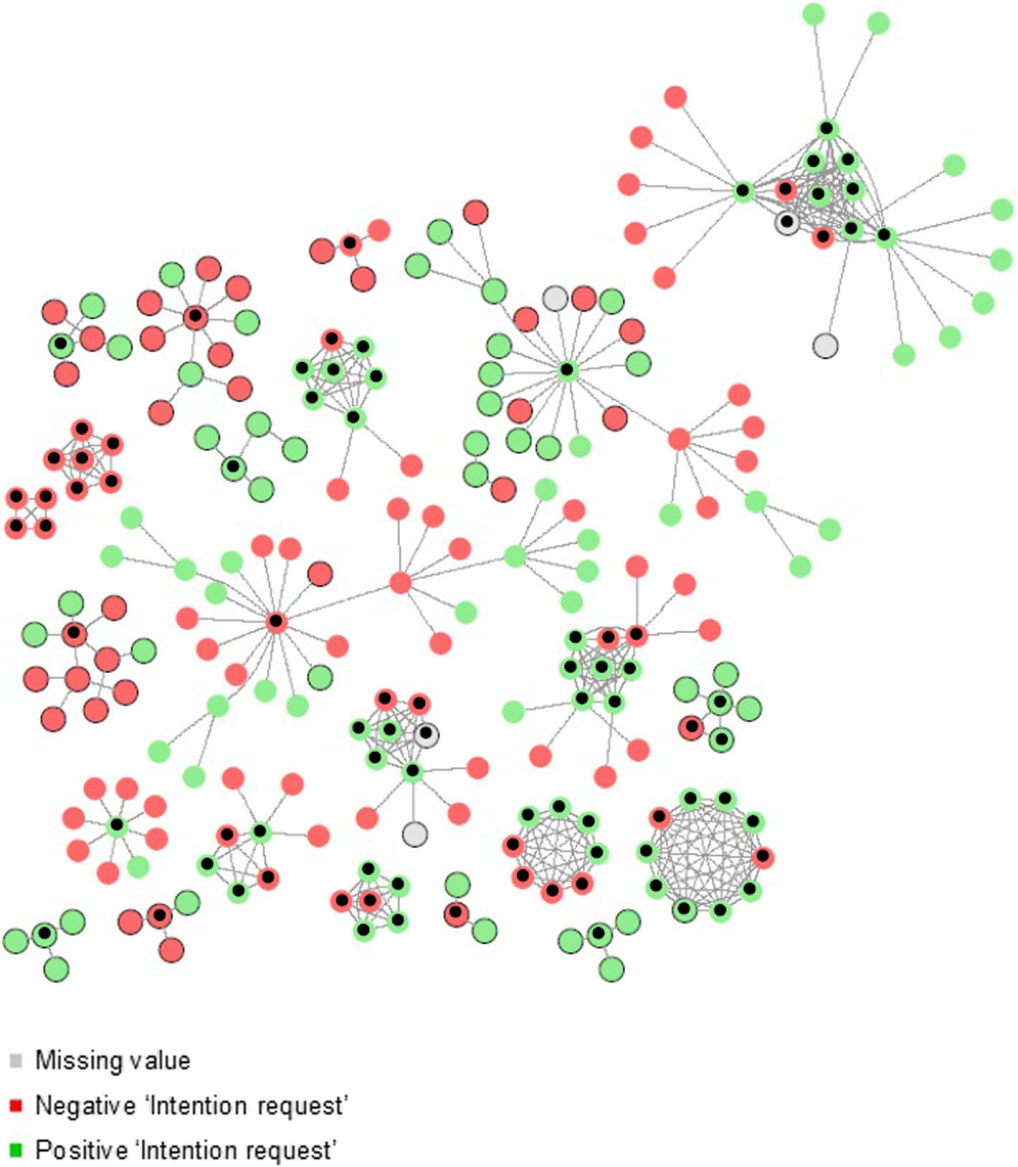
Screening intention among Moroccan immigrants (‘Intention request’). *Those recruited offline are presented as nodes with a transparent border, those recruited online are presented as nodes with a black border, and seeds are nodes with a black dot in the centre*

The maximum number of waves was four. Of the 24 clusters, there were eight with two or more waves. The largest cluster consisted of 35 participants (see Fig. 3). We obtained 390 recruiter-recruitee pairs: 154 pairs had a positive screening intention, 68 had a negative screening intention, 132 had a discordant screening intention, and for 36 pairs one or both individual(s) did not report their screening intention and were, therefore, not included in further analyses (see Table S3). Pairs with a negative intention more often had a Berber identity and were generally younger.

### Tie in relation to screening intention

Having a tie was associated with having the same screening intention and the association was not different for strong family ties compared to other ties (Model I-II), even after adjustment for covariates (Model III-IV) (see Table 2 in Additional file 1). Model IV provided the highest value of the log likelihood and, thus, performed the best. In this model, an OR of 1.70 [95% CI 1.15–2.51] was found for the association of having a tie on having the same screening intention. The greater the extent to which individuals believed that “screening gives clarity”, the higher the odds of having the same screening intention with an OR of 1.99 [95% CI 1.03–3.86]. Having the same educational level was negatively associated with having the same screening intention (OR 0.96 [95% CI 0.92–0.99]).

### Regrouping of screening intention

Of the 154 pairs with a positive screening intention, having a tie was associated with having the same *positive* screening intention with an OR of 1.56 [95% CI 1.11–2.17] (see Table S4). This indicates that positive intentions on screening cluster within social networks. Same response on fatalism (OR 1.90 [95% CI 1.12–3.21]), same response on “screening gives clarity” (OR 4.21 [95% CI 1.25–14.18]), and same response on self-efficacy (OR 2.18 [95% CI 1.05–4.49]) were all associated with having the same *positive* screening intention. The higher a pair perceived the risk of having chronic HBV, the higher the odds of having the same *positive* screening intention (OR 1.23 [95% CI 1.01–1.50]).

Of the 68 pairs with a negative screening intention, having a tie was not associated with having the same *negative* screening intention (OR 1.23 [0.97–2.09], see Table S5). Having the same response on fatalism and on self-efficacy decreased the odds of having the same *negative* screening intention with 0.69 [95% CI 0.53–0.89] and 0.57 [95% CI 0.36–0.90], respectively. The higher a pair perceived the risk of having chronic HBV, the lower the odds of having the same *negative* screening intention with 0.77 [95% CI 0.61–0.98], consistent with results of those having the same *positive* screening intention.

Ties also seem to reinforce the intention to participate in screening for a maximum up to EUR 70 (‘Intention 70’) (see Table S2). Woman-woman and man-woman pairs appeared to more often have the same screening intention in comparison to man-man pairs. The results of the analyses for outcome measure “Intention 70” can be found in Tables S2, S4, and S5 in the Supplementary information (Additional file 2).

## Discussion

We collected empirical data to study screening intentions in social networks of Moroccan immigrants and their offspring. Having a tie was associated with having the same intention to request a HBV test, as well as with the intention to participate in screening for a maximum compensation of up to EUR 70. By making use of respondent-driven sampling (RDS), we had the advantage of studying behaviour from socially interconnected individuals (i.e. we sampled both recruiters and recruitees) rather than considering responses reported by recruiters using only the persons in their own social network (i.e. ego-centric networks) as normally done in the literature [29].

A positive screening intention was clustered within the sampled social networks, while we found no indication of clustering for negative screening intention. The latter may be due to a limited number of sampled pairs with a negative screening intention (*n* = 68). In our study, pairs with a negative intention were younger and more often had a Berber identity (see Table S3). However, our logistic regression models did not indicate an association of these demographics with screening intention, but showed associations with determinants of individual screening intention only, which were also seen for pairs with a positive intention. Our findings suggest that interventions aimed at promoting screening participation may have benefits in the social group, beyond the individuals directly reached by these interventions, although we do not yet have a thorough understanding whether this is due to social influence. Testing this new hypothesis in an experimental setting is a future research direction.

By gradually including covariates in the model, we learned that having the same educational level had a negative association with having the same screening intention, which might be due to collinearity with some of the factors added in the final model. We also found that the more a pair thinks “screening gives clarity”, the higher the odds of having the same screening intention, which is consistent with previous work where this determinant was found as facilitator for intending to request a HBV test [12].

Pairs of woman-woman and man-woman had more often the same screening intention compared to man-man pairs for the intention to participate in screening for a maximum compensation of up to EUR 70 (‘Intention 70’). Pairs of the opposite sex will most likely be spouses or family members, since the Islam does not permit close social relationships with the opposite sex. Thus, spouses, family members of the opposite sex, and women more often have the same screening intention compared to men–men relationships. These findings suggest that we should not only focus on the individual when investigating (determinants of) screening behaviour, but incorporate screening behaviour of social contacts depending on sex (e.g. female spouses or friends) as well. It is necessary to approach decisions to screen (or not screen) not only from an individual perspective, but also consider the particular community in which target populations are immersed. However, whether and to what extent individuals influence each other (potentially leading to the same screening intention) should be studied using a more experimental treatment of individuals or by following social relations longitudinally. Such research could further direct on how to target communication strategies to enhance HBV screening participation.

Our results are consistent with previous work on a wide variety of behaviours and traits in social networks [42–44], such as obesity [19, 45], smoking [20, 46], happiness [25], and vaccination and cancer screening participation [47–49]. In our study, having a tie was shown to play a role in the intention to screen among those with a positive screening intention. This is partly in line with previous research, where researchers found social clustering of vaccine-refusers [50]. Stronger associations among pairs of women (found for ‘Intention 70’) were also observed for smoking in earlier research [46]. This is possibly because women engage in stronger relationships with a higher level of intimacy and reciprocity [51, 52]. Moreover, consistent with what we found for ‘Intention 70’, Christakis et al. found the highest decrease in a person’s chance of smoking when a spouse quits smoking in comparison to siblings and friends quitting [20].

It is important to recognize the limitations of the presented data. First, we assumed that participants recruited at the same venue had a tie, which may have caused an invalid overrepresentation of the variable tie. We assessed whether this was reasonable by including an interaction variable (“tie x RDS/venue ties”) in our final model. No association with our dependent variable was found (data not shown), which makes our assumption plausible. Second, we obtained a limited sample size and possibly a selective group, as we reached only a maximum of four waves and no Moroccans living in the north of the Netherlands (see Fig. S1). A larger sample size with a better geographic coverage would provide stronger evidence to generalise our findings. Nevertheless, this study provides empirical data on screening intention within social networks among a hard-to-reach population, namely Moroccan immigrants in the Netherlands. Potential reasons for individuals to refuse participation in our study were HBV-associated shame and stigma, and language barriers. To overcome these issues, we focused the questionnaire on the predominance of mother-to-child HBV transmission, and provided the option of having a face-to-face interview (at visited community venues) or a telephone interview, in either Berber or Moroccan-Arabic. Third, since participants received an incentive whenever they recruited three individuals, we did not stimulate recruitment of their complete social network and only investigated part of this network. This recruitment restriction in combination with having sampled only a maximum of four waves could have affected our results. Moreover, RDS recruitment is biased. Participants tend to invite the “right people” (eligible and/or reliable) that they believe would accept the invitation for the questionnaire or those that they feel will benefit from the questionnaire [53]. However, we did observe that participants recruited along different types of ties (e.g., family members, friends, acquaintances, workmates), which might have increased the diversity of intentions and traits in our sample. Furthermore, we investigated screening intention rather than actual behaviour (i.e. screening participation). Since previous research reported an observed discrepancy between intention and participation [54, 55], future research should also investigate screening participation within social networks to assess potential discrepancies. Additionally, our study population included more females and was higher educated in comparison to what was reported in the 2015 sample by Statistics Netherlands [12]. This bias is likely to be reduced by including gender and educational level in Models III and IV. Finally, because of the cross-sectional design of our study, we only captured a snapshot of individuals’ screening intention and did not study changes over time. Our data did not allow us to identify the underlying mechanism of the observed clustering of a positive screening intention. Whether this clustering is due to social influence has yet to be studied. If so, it might be necessary to set up so-called “induction interventions” where peer-to-peer interactions are stimulated or forced to create cascades in information/behavioural diffusion using word-of-mouth, RDS, or network outreach (i.e. seeds recruit members of their personal networks to participate in an intervention together) [31].

## Conclusions

Out of all the variables considered in this study including sociodemographic characteristics, having a tie was the most important one in terms of one’s screening intention. These findings emphasise the need to take the social network of individuals into account when studying individual behaviour concerning screening participation. The next step is to investigate if and how peers and/or other community members can be used to disseminate information for informed decision-making regarding screening programmes, such as chronic HBV screening.

## Supplementary information

**Additional file 1: Table 2.** Logistic regression analyses of having the same screening intention (‘Intention request’) in relation to having a tie.

**Additional file 2: Table S1.** Overview of variables measured by the questionnaire. **Table S2.** Logistic regression analyses of having the same screening intention (‘Intention 70’) in relation to having a tie. **Table S3.** Sample characteristics for pairs with a discordant, positive, and negative screening intention. **Table S4.** Logistic regression analyses of having the same positive screening intention (‘Intention request’) in relation to having a tie. **Table S5.** Logistic regression analyses of having the same negative screening intention (‘Intention request’) in relation to having a tie. **Figure S1.** The geographical distribution of our participants [56]

## Data Availability

The datasets generated and/or analysed during the current study are not publicly available due to privacy reasons, but are available from the corresponding author on reasonable request.

## Abbreviations

GP: General practitioner
HBM: Health Belief Model
HBsAg: Hepatitis B surface antigen
HBV: Hepatitis B virus
RDS: Respondent-driven sampling
RIVM: National Institute for Public Health and the Environment
SR: Self-reported
TPB: Theory of Planned Behaviour
WHO: World Health Organization
