# Supplementary material for: Clustering of chronic hepatitis B screening intentions in social networks of Moroccan immigrants in the Netherlands
Source: BMC Public Health. 2020 Mar 17;20:344. doi: 10.1186/s12889-020-8438-x (PMC7077096; doi:10.1186/s12889-020-8438-x)
Supplement: Supplementary file 1 — Additional file 1: Table 2. Logistic regression analyses of having the same screening intention (‘Intention request’) in relation to having a tie. [file 12889_2020_8438_MOESM1_ESM.docx]

1 **Table 2. Logistic regression analyses of having the same screening intention (‘Intention request’) in relation to having a tie**

|  | **Model I**  **Npotential = 25651 (Log likelihood = -**  **17767)** | | **Model II Npotential = 25493**  **(Log likelihood = -17657)** | | **Model III Npotential = 19175**  **(Log likelihood = -13226)** | | **Model IV Npotential = 18019**  **(Log likelihood = -12317)** | |
| --- | --- | --- | --- | --- | --- | --- | --- | --- |
| **Variables** | **OR [95%**  **CI]** | **p-value** | **OR [95% CI]** | **p-value** | **OR [95%**  **CI]** | **p-value** | **OR [95% CI]** | **p-value** |
| Tie | 1.71 | 0.001* | 1.70 | 0.001* | 1.70 | 0.003* | 1.70 | 0.008* |
|  | [1.25 – |  | [1.25 – 2.32] |  | [1.20 – 2.40] |  | [1.15 – 2.51 |  |
|  | 2.34] |  |  |  |  |  |  |  |
| *Close family* |  |  | 0.97 | 0.222 | 0.97 | 0.495 | 0.99 | 0.816 |
| *relationship* |  |  | [0.92 – 1.02] |  | [0.88 – 1.06] |  | [0.90 – 1.08] |  |
| Same gender: |  |  |  |  |  |  |  |  |
| W-W vs M-M |  |  |  |  | 1.09 | 0.172 | 1.07 | 0.254 |
|  |  |  |  |  | [0.96 – 1.24] |  | [0.95 – 1.20] |  |
| Same gender: |  |  |  |  |  |  |  |  |
| M-W vs M-M |  |  |  |  | 1.02 | 0.789 | 1.03 | 0.656 |
|  |  |  |  |  | [0.87 – 1.20] |  | [0.89 – 1.19] |  |

| Mean age |  |  |  |  | 1.00  [0.99 – 1.00] | 0.284 | 1.00  [0.99 – 1.00] | 0.319 |
| --- | --- | --- | --- | --- | --- | --- | --- | --- |
| Difference in age |  |  |  |  | 1.00  [1.00 – 1.01] | 0.370 | 1.00  [1.00 – 1.01] | 0.316 |
| Same country of birth:  NL-NL vs MR-MR |  |  |  |  | 0.85  [0.67 – 1.09] | 0.199 | 0.89  [0.76 – 1.05] | 0.170 |
| Same country of birth:  NL-MR vs MR-  MR |  |  |  |  | 0.82  [0.64 – 1.07] | 0.143 | 0.86  [0.70 – 1.04] | 0.127 |
| Same  educational level |  |  |  |  | 0.97  [0.91 – 1.04] | 0.402 | 0.96  [0.92 – 0.99] | 0.028* |
| Mean  educational level |  |  |  |  | 0.99  [0.95 – 1.03] | 0.634 | 0.99  [0.95 – 1.04] | 0.780 |
| Same response  on fatalism |  |  |  |  | 1.29  [0.90 – 1.86] | 0.170 | 1.32  [0.90 – 1.93] | 0.162 |

| Mean value on |  |  |  |  | 1.07 | 0.341 | 1.05 | 0.300 |
| --- | --- | --- | --- | --- | --- | --- | --- | --- |
| fatalism |  |  |  |  | [0.93 – 1.25] |  | [0.95 – 1.16] |  |
| Same response |  |  |  |  |  |  | 2.36 | 0.087 |
| on “screening |  |  |  |  |  |  | [0.88 – 6.30] |  |
| gives clarity” |  |  |  |  |  |  |  |  |
| Mean value on |  |  |  |  |  |  | 1.99 | 0.042* |
| “screening gives |  |  |  |  |  |  | [1.03 – 3.86] |  |
| clarity” |  |  |  |  |  |  |  |  |
| Same response |  |  |  |  |  |  | 1.26 | 0.097 |
| on “screening not |  |  |  |  |  |  | [0.96 – 1.65] |  |
| needed if no |  |  |  |  |  |  |  |  |
| symptoms” |  |  |  |  |  |  |  |  |
| Mean value on |  |  |  |  |  |  | 1.06 | 0.447 |
| “screening not |  |  |  |  |  |  | [0.91 – 1.24] |  |
| needed if no |  |  |  |  |  |  |  |  |
| symptoms” |  |  |  |  |  |  |  |  |
| Same response |  |  |  |  |  |  | 1.21 | 0.307 |
| on self-efficacy |  |  |  |  |  |  | [0.84 – 1.73] |  |

| Mean value on  self-efficacy |  |  |  |  |  |  | 1.20  [0.95 – 1.50] | 0.121 |
| --- | --- | --- | --- | --- | --- | --- | --- | --- |
| Same response on risk  perception |  |  |  |  |  |  | 1.10  [0.97 – 1.25] | 0.146 |
| Mean value on  risk perception |  |  |  |  |  |  | 1.02  [0.96 – 1.09] | 0.449 |

2 *p < 0.05

1. We used the variance estimator proposed by Cameron et al, which is a robust method to correct standard errors for multi-way
2. clustering (41).
