# Supplementary material for: Clustering of chronic hepatitis B screening intentions in social networks of Moroccan immigrants in the Netherlands
Source: BMC Public Health. 2020 Mar 17;20:344. doi: 10.1186/s12889-020-8438-x (PMC7077096; doi:10.1186/s12889-020-8438-x)
Supplement: Supplementary file 2 — Additional file 2: Table S1. Overview of variables measured by the questionnaire. Table S2. Logistic regression analyses of having the same screening intention (‘Intention 70’) in relation to having a tie. Table S3. Sample characteristics for pairs with a discordant, positive, and negative screening intention. Table S4. Logistic regression analyses of having the same positive screening intention (‘Intention request’) in relation to having a tie. Table S5. Logistic regression analyses of having the same negative screening intention (‘Intention request’) in relation to having a tie. Figure S1. The geographical distribution of our participants [56] [file 12889_2020_8438_MOESM2_ESM.docx]

***Supplementary information: Clustering of chronic hepatitis B screening intentions in social networks of Moroccan immigrants in the Netherlands***

*Nora Hamdiui^a,b^, Vincent Buskens^c^, Jim E. van Steenbergen^a,d^, Mirjam E. E. Kretzschmar^b,e^, Luis E. C. Rocha^f^, Anna E. Thorson^g^, Aura Timen^a,h^, Albert Wong^i^, Maria van den Muijsenbergh^j,k^, Mart L. Stein^a^*

**Table S1. Overview of variables measured by the questionnaire**

| *Constructs* | *Variables* | *Short variable descriptions* | *Options* |
| --- | --- | --- | --- |
| *Perceived risk* | Risk without noticing | Risk having HBV without noticing | Low  Quite low  Average  Quite high  High  I do not know |
|  | Risk infecting someone | Risk of infecting someone with HBV | Low  Quite low  Average  Quite high  High  I do not know |
| *Perceived severity of disease* | Severity feeling good | HBV is no problem when feeling good | Yes  No  I do not know |
|  | Severity serious disease | HBV is a serious disease | Yes  No  I do not know |
| *Stigma regarding HBV* | Stigma friends | Others not wanting to be friends when having HBV | Yes  No  I do not know |
|  | Stigma respect | Others less respect when having HBV | Yes  No  I do not know |
|  | Stigma comfort | Others feeling uncomfortable when having HBV | Yes  No  I do not know |
| *Shame regarding HBV* | Shame others | Feeling ashamed when others know HBV status | Yes  No  I do not know |
|  | Shame guilty | Feeling guilty having HBV | Yes  No  I do not know |
|  | Shame fear | Feeling feared having HBV | Yes  No  I do not know |
|  | Shame disappointment | Feeling disappointed having HBV | Yes  No  I do not know |
|  | Shame not caring | Do not care if others know HBV status | Yes  No  I do not know |
| *Perceived*  *self-efficacy* | Self-efficacy | Able to decide HBV screening participation | Yes  No  I do not know |
| *Social influence* | Social influence friends | Will test myself if friend recommends | Yes  No  I do not know |
|  | Social influence imam | Will test myself if imam recommends | Yes  No  I do not know |
| *Perceived benefits* | Benefit own health | HBV screening good for my health | Yes  No  I do not know |
|  | Benefit health others | HBV screening good for health others | Yes  No  I do not know |
|  | Benefit clarity | HBV screening gives clarity | Yes  No  I do not know |
| *Perceived barriers* | Barrier too much time | HBV screening takes too much time | Yes  No  I do not know |
|  | Barrier important | HBV screening not important | Yes  No  I do not know |
|  | Barrier not having symptoms | HBV screening not needed if no symptoms | Yes  No  I do not know |
|  | Barrier trusting Allah | HBV screening not needed only trust Allah | Yes  No  I do not know |
| *Knowledge on HBV* | Knowledge on HBV | I know nothing about HBV  HBV is an infectious disease  HBV can cause liver cancer  Someone who is looking healthy and feeling good cannot infect others with HBV | True  False |
| *Moroccan Arabic or Berber identity* | Moroccan-Arabic or Berber identity | - | Moroccan-Arabic  Berber |
| *Speaking Dutch* | Speaking Dutch | The ability to speak Dutch | Yes  No |
| *Knowing someone having HBV* | Knowing someone having HBV | Knowing someone having HBV | Yes  No  I do not know |
| *Tested for HBV* | Tested for HBV | Tested for HBV, self-reported | Yes  No  I do not know |
| *Vaccinated against HBV* | Vaccinated against HBV | Vaccinated against HBV, self-reported | Yes  No  I do not know |

**Table S2. Logistic regression analyses of having the same screening intention (‘Intention 70’) in relation to having a tie**

|  | **Model I**  **N_potential_ = 23436**  **(Log likelihood = -16233)** | | **Model II**  **N_potential_ = 23288**  **(Log likelihood = -16130)** | | **Model III**  **N_potential_ = 17831**  **(Log likelihood = -12349882)** | | **Model IV**  **N_potential_ = 17085**  **(Log likelihood = -11832)** | | |
| --- | --- | --- | --- | --- | --- | --- | --- | --- | --- |
| **Variables** | **OR [95% CI]** | **p-value** | **OR [95% CI]** | **p-value** | **OR [95% CI]** | **p-value** | **OR [95% CI]** | **p-value** |  |
| Tie | 1.69  [1.13 – 2.53] | 0.010* | 1.71  [1.15 – 2.55] | 0.008* | 1.71  [1.11 – 2.65] | 0.016* | 1.62  [1.09 – 2.42] | 0.017* |  |
| Type of tie  *Close family relationship yes/no* |  |  | 1.01  [0.97 – 1.05] | 0.624 | 1.03  [0.97 – 1.11] | 0.335 | 1.05  [0.99 – 1.11] | 0.084 |  |
| Same gender:  W-W vs M-M |  |  |  |  | 1.03  [0.92 – 1.15] | 0.615 | 1.03  [1.00 – 1.07] | 0.076 |  |
| Same gender:  M-W vs M-M |  |  |  |  | 1.04  [1.01 – 1.08] | 0.019* | 1.05  [1.03 – 1.07] | <0.001* |  |
| Mean age |  |  |  |  | 1.00  [1.00 – 1.00] | 0.916 | 1.00  [0.99 – 1.01] | 0.853 |  |
| Difference in age |  |  |  |  | 1.00  [1.00 – 1.00] | 0.899 | 1.00  [0.99 – 1.01] | 0.974 |  |
| Same country of birth:  NL-NL vs MR-MR |  |  |  |  | 1.01  [0.77 – 1.32] | 0.965 | 0.97  [0.69 – 1.36] | 0.850 |  |
| Same country of birth:  NL-MR vs MR-MR |  |  |  |  | 1.02  [0.88 – 1.17] | 0.840 | 0.99  [0.84 – 1.18] | 0.928 |  |
| Same educational level |  |  |  |  | 1.00  [0.92 – 1.08] | 0.936 | 0.99  [0.91 – 1.07] | 0.789 |  |
| Mean educational level |  |  |  |  | 1.00  [0.91 – 1.11] | 0.922 | 1.00  [0.91 – 1.10] | 0.953 |  |
| Same response on fatalism |  |  |  |  | 0.98  [0.94 – 1.02] | 0.257 | 0.97  [0.92 – 1.02] | 0.224 |  |
| Mean value on fatalism |  |  |  |  | 1.00  [0.87 – 1.14] | 0.962 | 1.00  [0.88 – 1.14] | 0.960 |  |
| Same response on “screening gives clarity” |  |  |  |  |  |  | 0.98  [0.94 – 1.02] | 0.295 |  |
| Mean value on “screening gives clarity” |  |  |  |  |  |  | 1.00  [0.85 – 1.18] | 0.995 |  |
| Same response on “screening not needed if no symptoms” |  |  |  |  |  |  | 1.04  [0.94 – 1.16] | 0.395 |  |
| Mean value on “screening not needed if no symptoms” |  |  |  |  |  |  | 1.00  [0.89 – 1.12] | 0.997 |  |
| Same response on self-efficacy |  |  |  |  |  |  | 1.03  [0.91 – 1.16] | 0.643 |  |
| Mean value on self-efficacy |  |  |  |  |  |  | 0.99  [0.90 – 1.10] | 0.864 |  |
| Same response on risk  perception |  |  |  |  |  |  | 1.18  [0.77 – 1.80] | 0.448 |  |
| Mean value on risk perception |  |  |  |  |  |  | 1.21  [0.99 – 1.47] | 0.059 |  |

*p < 0.05

We used the variance estimator proposed by Cameron et al, which is a robust method to correct standard errors for multi-way clustering (41).

**Table S3. Sample characteristics for pairs with a discordant, positive, and negative screening intention**

| *Characteristic* | | *Discordant pairs*  *(132 pairs with 124 unique individuals)* | *Positive pairs*  *(154 pairs with 104 unique individuals)* | *Negative pairs*  *(68 pairs with 67 unique individuals)* |
| --- | --- | --- | --- | --- |
| *Offline or online questionnaire participation* | Offline  Online  *Missing value* | 90 (72.6)  34 (27.4)  0 (0) | 69 (67.6)  33 (32.4)  0 (0) | 45 (67.2)  22 (32.8)  0 (0) |
| *Country of birth* | Morocco  The Netherlands  *Missing value* | 75 (60.5)  49 (39.5)  0 (0) | 69 (67.6)  33 (32.4)  0 (0) | 41 (61.2)  26 (38.8)  0 (0) |
| *Moroccan-Arabic or Berber identity* | Arabic  Berber  *Missing value* | 51 (41.1)  72 (58.1)  1 (0.8) | 42 (41.2)  60 (58.8)  0 (0) | 20 (29.9)  46 (68.7)  1 (1.5) |
| *Gender* | Man  Woman  *Missing value* | 35 (28.2)  89 (71.8)  0 (0) | 29 (28.4)  73 (71.6)  0 (0) | 21 (31.3)  46 (68.7)  0 (0) |
| *Age group* | 16 – 25 years  26 – 35 years  36 – 45 years  46 – 55 years  56 – 65 years  66 years and older  *Missing value* | 23 (18.5)  17 (13.7)  31 (25.0)  22 (17.7)  17 (13.7)  7 (5.6)  7 (5.6) | 14 (13.7)  17 (16.7)  22 (21.6)  28 (27.5)  11 (10.8)  7 (6.9)  3 (2.9) | 16 (23.9)  6 (9.0)  18 (26.9)  11 (16.4)  10 (14.9)  2 (3.0)  4 (6.0) |
| *Educational level* | No official education or primary school  Secondary school  Vocational education  Higher education  *Missing value* | 32 (25.8)  21 (16.9)  28 (22.6)  39 (31.5)  4 (3.2) | 28 (27.5)  16 (15.7)  24 (23.5)  33 (32.4)  1 (1.0) | 13 (19.4)  19 (28.4)  15 (22.4)  18 (26.9)  2 (3.0) |
| *Speaking Dutch (SR)* | Yes  No  *Missing value* | 7 (5.6)  116 (93.5)  1 (0.8) | 6 (5.9)  96 (94.1)  0 (0) | 4 (6.0)  62 (92.5)  1 (1.5) |
| *Knowledge on HBV* | No  Limited  Sufficient  *Missing value* | 45 (36.3)  55 (44.4)  24 (19.4)  0 (0) | 42 (41.2)  38 (37.3)  22 (21.6)  0 (0) | 29 (43.3)  25 (37.3)  13 (19.4)  0 (0) |
| *HBV in family or friends* | Yes  No  I do not know  *Missing value* | 33 (26.6)  80 (64.5)  11 (8.9)  0 (0) | 29 (28.4)  58 (56.9)  15 (14.7)  0 (0) | 13 (19.4)  45 (67.2)  9 (13.4)  0 (0) |
| *Tested for HBV (SR)* | Yes  No  I do not know  *Missing value* | 31 (25.0)  84 (67.7)  8 (6.5)  1 (0.8) | 20 (19.6)  69 (67.6)  12 (11.8)  1 (1.0) | 13 (19.4)  47 (70.1)  7 (10.4)  0 (0) |
| *Vaccinated against HBV (SR)* | Yes  No  I do not know  *Missing value* | 39 (31.5)  40 (32.3)  45 (36.3)  0 (0) | 29 (28.4)  34 (33.3)  39 (38.2)  0 (0) | 20 (29.9)  20 (29.9)  27 (40.3)  0 (0) |

Data are reported as number of participants (%).

SR: Self-reported.**Table S4. Logistic regression analyses of having the same positive screening intention (‘Intention request’) in relation to having a tie**

|  | **Model I**  **N_potential_ = 25887**  **(Log likelihood =-15451)** | | **Model II**  **N_potential_ = 25729**  **(Log likelihood = -15306)** | | **Model III**  **N_potential_ = 19247**  **(Log likelihood = -11218)** | | **Model IV**  **N_potential_ = 18090**  **(Log likelihood = -9535)** | | |
| --- | --- | --- | --- | --- | --- | --- | --- | --- | --- |
| **Variables** | **OR [95% CI]** | **p-value** | **OR [95% CI]** | **p-value** | **OR [95% CI]** | **p-value** | **OR [95% CI]** | **p-value** |  |
| Tie | 2.04  [1.50 – 2.76] | < 0.001* | 2.00  [1.49 – 2.69] | < 0.001* | 1.57  [1.20 – 2.06] | 0.001* | 1.56  [1.11 – 2.17] | 0.010* |  |
| Type of tie  *Close family relationship yes/no* |  |  | 0.80  [0.55 – 1.19] | 0.271 | 0.82  [0.50 – 1.37] | 0.453 | 0.86  [0.53 – 1.41] | 0.558 |  |
| Same gender:  W-W vs M-M |  |  |  |  | 2.05  [0.82 – 5.12] | 0.125 | 1.66  [0.68 – 4.06] | 0.267 |  |
| Same gender:  M-W vs M-M |  |  |  |  | 1.45  [1.04 – 2.03] | 0.031* | 1.31  [0.93 – 1.83] | 0.119 |  |
| Mean age |  |  |  |  | 0.99  [0.96 – 1.03] | 0.722 | 1.00  [0.97 – 1.04] | 0.986 |  |
| Difference in age |  |  |  |  | 1.01  [0.99 – 1.02] | 0.459 | 1.01  [0.99 – 1.02] | 0.507 |  |
| Same country of birth:  NL-NL vs MR-MR |  |  |  |  | 0.35  [0.13 – 0.95] | 0.039* | 0.51  [0.18 – 1.47] | 0.213 |  |
| Same country of birth:  NL-MR vs MR-MR |  |  |  |  | 0.55  [0.28 – 1.06] | 0.074 | 0.68  [0.34 – 1.33] | 0.257 |  |
| Same educational level |  |  |  |  | 1.00  [0.90 – 1.11] | 0.995 | 0.99  [0.90 – 1.08] | 0.799 |  |
| Mean educational level |  |  |  |  | 0.97  [0.72 – 1.30] | 0.819 | 1.05  [0.77 – 1.45] | 0.751 |  |
| Same response on fatalism |  |  |  |  | 1.95  [1.22 – 3.11] | 0.005* | 1.90  [1.12 – 3.21] | 0.017* |  |
| Mean value on fatalism |  |  |  |  | 1.62  [0.66 – 3.97] | 0.293 | 1.51  [0.61 – 3.73] | 0.376 |  |
| Same response on “screening gives clarity” |  |  |  |  |  |  | 4.21  [1.25 – 14.18] | 0.020* |  |
| Mean value on “screening gives clarity” |  |  |  |  |  |  | 0.63  [0.23 – 1.72] | 0.362 |  |
| Same response on “screening not needed if no symptoms” |  |  |  |  |  |  | 1.52  [0.96 – 2.42] | 0.077 |  |
| Mean value on “screening not needed if no symptoms” |  |  |  |  |  |  | 1.74  [0.38 – 3.32] | 0.095 |  |
| Same response on self-efficacy |  |  |  |  |  |  | 2.18  [1.05 – 4.49] | 0.035* |  |
| Mean value on self-efficacy |  |  |  |  |  |  | 1.79  [0.76 – 4.21] | 0.095 |  |
| Same response on risk  perception |  |  |  |  |  |  | 0.96  [0.85 – 1.08] | 0.463 |  |
| Mean value on risk perception |  |  |  |  |  |  | 1.23  [1.01 – 1.50] | 0.043* |  |

*p < 0.05

We used the variance estimator proposed by Cameron et al, which is a robust method to correct standard errors for multi-way clustering (41).**Table S5. Logistic regression analyses of having the same negative screening intention (‘Intention request’) in relation to having a tie**

|  | **Model I**  **N_potential_ = 25889**  **(Log likelihood = -13335)** | | **Model II**  **N_potential_ = 25731**  **(Log likelihood = -13280)** | | **Model III**  **N_potential_ = 19252**  **(Log likelihood = -9238)** | | **Model IV**  **N_potential_ = 18093**  **(Log likelihood = -8226)** | | |
| --- | --- | --- | --- | --- | --- | --- | --- | --- | --- |
| **Variables** | **OR [95% CI]** | **p-value** | **OR [95% CI]** | **p-value** | **OR [95% CI]** | **p-value** | **OR [95% CI]** | **p-value** |  |
| Tie | 0.81  [0.47 – 1.37] | 0.426 | 0.82  [0.48 – 1.38] | 0.455 | 1.18  [0.66 – 2.12] | 0.576 | 1.23  [0.97 – 2.09] | 0.437 |  |
| Type of tie  *Close family relationship yes/no* |  |  | 1.26  [0.86 – 1.85] | 0.239 | 1.18  [0.74 – 1.90] | 0.483 | 1.20  [0.74 – 1.94] | 0.470 |  |
| Same gender:  W-W vs M-M |  |  |  |  | 0.53  [0.31 – 1.33] | 0.178 | 0.65  [0.26 – 1.62] | 0.356 |  |
| Same gender:  M-W vs M-M |  |  |  |  | 0.73  [0.46 – 1.16] | 0.184 | 0.81  [0.52 – 1.28] | 0.377 |  |
| Mean age |  |  |  |  | 1.00  [0.97 – 1.04] | 0.787 | 0.99  [0.95 – 1.04] | 0.735 |  |
| Difference in age |  |  |  |  | 1.00  [0.98 – 1.01] | 0.566 | 1.00  [0.98 – 1.01] | 0.654 |  |
| Same country of birth:  NL-NL vs MR-MR |  |  |  |  | 2.89  [0.94 – 8.86] | 0.063 | 1.66  [0.48 – 5.78] | 0.423 |  |
| Same country of birth:  NL-MR vs MR-MR |  |  |  |  | 1.78  [0.94 – 3.38] | 0.079 | 1.32  [0.66 – 2.61] | 0.433 |  |
| Same educational level |  |  |  |  | 0.95  [0.90 – 1.01] | 0.110 | 1.05  [0.89 – 1.02] | 0.177 |  |
| Mean educational level |  |  |  |  | 1.04  [0.75 – 1.45] | 0.794 | 0.90  [0.63 – 1.29] | 0.580 |  |
| Same response on fatalism |  |  |  |  | 0.63  [0.52 – 0.77] | <0.001* | 0.69  [0.53 – 0.89] | 0.004* |  |
| Mean value on fatalism |  |  |  |  | 0.63  [0.25 – 1.57] | 0.322 | 0.72  [0.32 – 1.60] | 0.420 |  |
| Same response on “screening gives clarity” |  |  |  |  |  |  | 0.92  [0.55 – 1.53] | 0.738 |  |
| Mean value on “screening gives clarity” |  |  |  |  |  |  | 2.47  [0.89 – 6.80] | 0.081 |  |
| Same response on “screening not needed if no symptoms” |  |  |  |  |  |  | 0.83  [0.66 – 1.04] | 0.109 |  |
| Mean value on “screening not needed if no symptoms” |  |  |  |  |  |  | 0.60  [0.34 – 1.08] | 0.089 |  |
| Same response on self-efficacy |  |  |  |  |  |  | 0.57  [0.36 – 0.90] | 0.016* |  |
| Mean value on self-efficacy |  |  |  |  |  |  | 0.77  [0.29 – 2.02] | 0.590 |  |
| Same response on risk  perception |  |  |  |  |  |  | 1.10  [0.89 – 1.37] | 0.374 |  |
| Mean value on risk perception |  |  |  |  |  |  | 0.77  [0.61 – 0.98] | 0.032* |  |

*p < 0.05

We used the variance estimator proposed by Cameron et al, which is a robust method to correct standard errors for multi-way clustering (41).


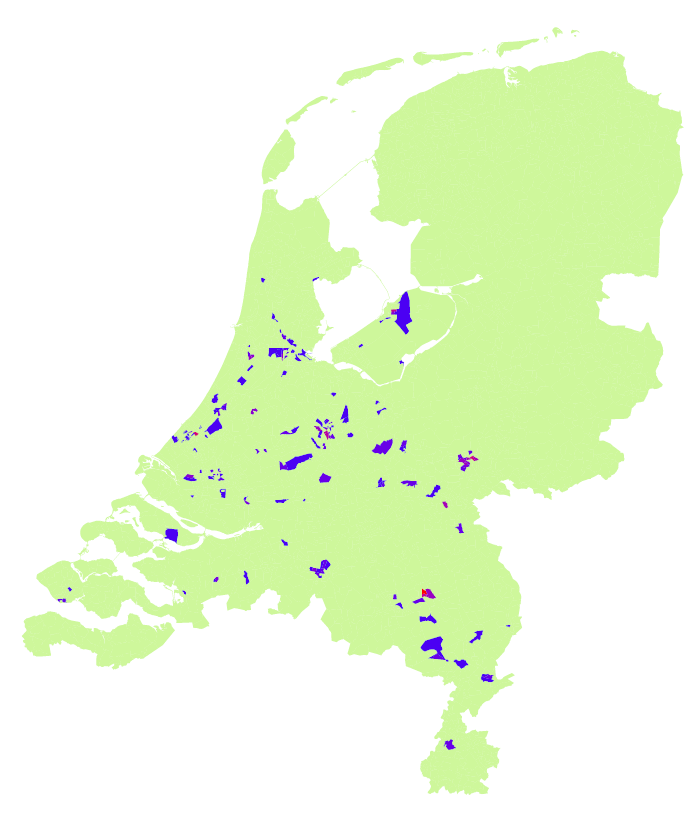


**Figure S1. The geographical distribution of our participants**

This map was created using R version 3.4.0 with a shapefile (.shp file) that was extracted from GADM, an online geographic database of global administrative areas, that is freely available for academic and other non-commercial use and allowed for academic publishing (57).
